# Ultrafast Myocardial Contrast Echocardiography for the Assessment of Coronary Artery Disease: First In-Human Study

**DOI:** 10.1161/CIRCIMAGING.124.017267

**Published:** 2024-10-02

**Authors:** Lasha Gvinianidze, Matthieu Toulemonde, Reinette Hampson, Biao Huang, Gabriel Bioh, Leigh-Ann Wakefield, Ashish Patel, Kiruba Rajan, Meng-Xing Tang, Roxy Senior

**Affiliations:** Northwick Park and St Mark’s Hospitals, London Northwest University Healthcare, Harrow, United Kingdom (L.G., R.H., G.B., L.-A.W., A.P., K.R., R.S.).; National Heart and Lung Institute, Imperial College and Royal Brompton Hospital, London, United Kingdom (L.G., G.B., R.S.).; Department of Bioengineering, Imperial College London, United Kingdom (M.T., B.H., M.-X.T.).

**Keywords:** coronary artery disease, high frame rate cardiac imaging, myocardial contrast echocardiography, perfusion, ultrafast ultrasound

Clinical myocardial contrast echocardiography (MCE) utilizes commercial microbubbles for myocardial perfusion assessment for the detection of obstructive coronary artery disease (OCAD). However, its accuracy is limited by the image quality.^1^ Development of transmission of nonfocused waves with digital beam forming allows images to be obtained with thousands of frames per second (FPS), compared with only 30 to 40 FPS by conventional MCE.^2^ High frame rate (HFR) or ultrafast ultrasound MCE due to its high temporal resolution can reduce noise and minimize motion artifacts, thus improving perfusion assessment as demonstrated in healthy volunteers.^3^ We thus proceeded to test the value of HFR MCE for the assessment of myocardial ischemia in patients with OCAD. The study was approved by the Institutional Review Committee and the patients gave informed consent.

The data supporting the findings of this study will be available on request from the authors. Twenty-five consecutive consenting eligible patients (age, 64; mean±9.9 SD; male, 18 and female, 7) underwent rest and vasodilator (dipyridamole: 22 patients and regadenoson: 3 patients) perfusion imaging using both conventional (Philips IE 33, Eindhoven, Holland) and HFR (Verasonics Vantage TM) techniques prospectively during intravenous infusion of ultrasound contrast agent (Sonovue, Bracco, Italy) using power modulation technique (multi-pulse with alternating amplitudes), low mechanical index (0.1) imaging with both modalities, in the 3 apical views. Of these, 20 patients had a high pretest probability of OCAD, based on the presence of inducible wall motion abnormality on clinical contrast-enhanced stress echocardiography. Five patients with a low pretest probability of OCAD and no inducible wall motion abnormality were in the control group. Of the 25 patients undergoing clinical stress echocardiography, 24 patients underwent exercise and 1 patient had dobutamine. Flash-replenishment imaging was performed to clear microbubbles from the myocardium with a transient increase in the mechanical index to 0.6 to 0.9 (flash), followed by low mechanical index to observe the rate of microbubble replenishment. Conventional MCE was performed with both real-time at 30 FPS as well as triggered (Tr) imaging whereas HFR was performed with only real-time imaging at 300 FPS. Conventional and HFR MCE were performed in the same sequence in all patients. An inducible perfusion defect was defined as present if the myocardium replenished after 2 seconds following the flash accompanied by subendocardial or transmural defect replenishing from subepicardium to subendocardium. The severity of perfusion abnormality was graded as 1 if the myocardium replenished between 2 and 5 seconds (mild-moderate) and 0 beyond 5 seconds (severe). The analysis was done using the 16-segment left ventricular model. The segments were accordingly assigned to each of the major vascular territories. The reviewers were blinded for the reads of the 2 imaging modalities.

Of the 20 patients with inducible ischemia on clinical stress echocardiography, 18 patients showed OCAD on coronary angiography. All patients with OCAD demonstrated inducible perfusion defects with both conventional and HFR MCE corresponding to OCAD territory. Both techniques correctly detected multivessel OCAD. However, the number of segments with inducible perfusion defects by HFR was larger than that with conventional MCE, 114 and 75, respectively (*P*<0.001) and were more intense, as shown in the Figure with examples from 3 patients with OCAD. In 9 (50%) out of 18 patients with OCAD, HFR MCE detected severe inducible perfusion defect compared with mild defect picked up by conventional MCE (*P*<0.001). Of the 2 patients with high pretest probability for CAD but no OCAD on angiography, both MCE techniques detected perfusion defects in distal LAD territories. Of the 5 patients with low pretest probability for OCAD, both MCE techniques detected no perfusion defects in 3 patients. One patient was found to have inducible apical perfusion defect by both techniques but without OCAD. The test was terminated in the fifth patient due to dipyridamole-induced side effects. Inter- and intraobserver reproducibility in 12 randomly selected patients showed concordance of 92% and 94% on a vascular territory basis, respectively.

**Figure.**
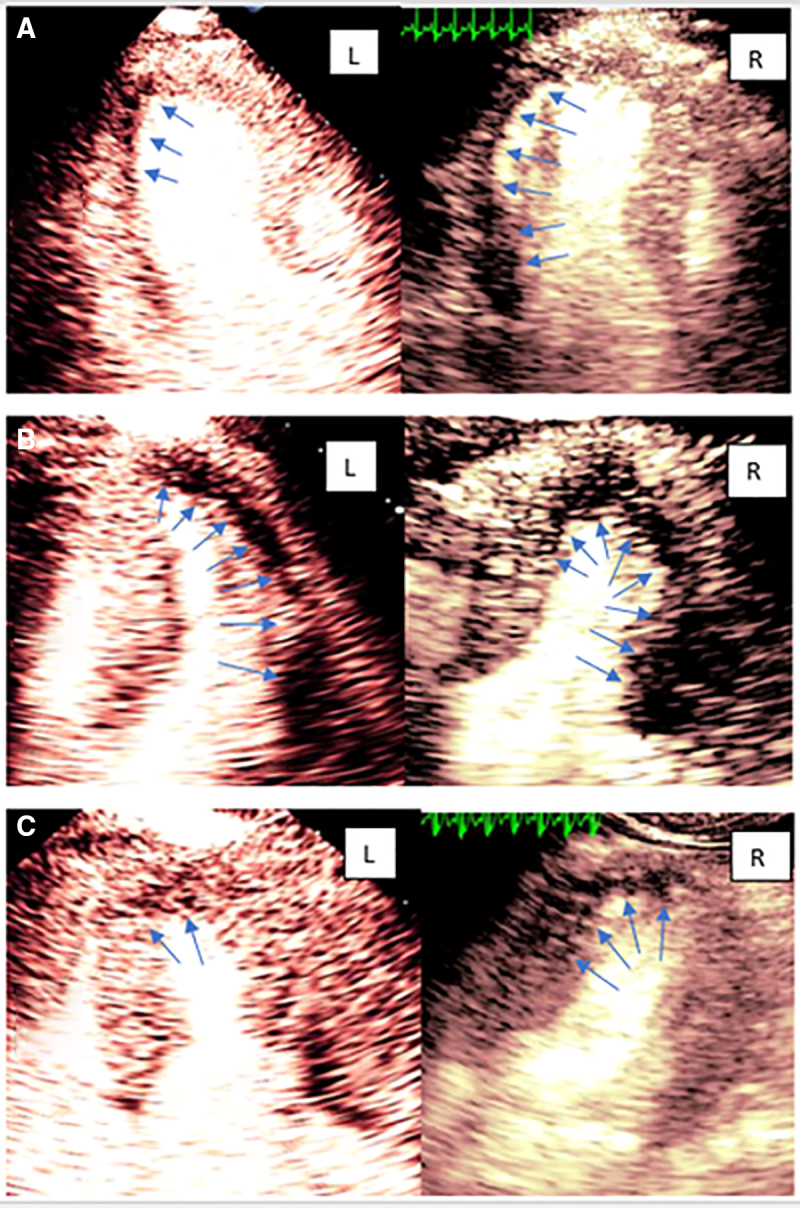
**Conventional myocardial contrast echocardiography (MCE; left) and high frame rate (HFR) MCE (right) end-systolic images. A**, Occluded single obtuse marginal artery (OM) artery. Three-chamber view: the arrows demonstrate perfusion defect within inferolateral wall. HFR image shows more extensive and intense perfusion defect with underlying wall thickening abnormality compared with conventional MCE in a patient with single vessel occluded large OM. **B**, Four-chamber view. HFR demonstrates moderate intensity perfusion defect of mid to distal inferoseptal and apex and severe defect of the anterolateral wall. Corresponding conventional MCE shows less extensive apical defect with a large anterolateral defect. The patient had mid left anterior descending artery (LAD) and a large caliber left circumflex artery (LCx) obstructive coronary artery disease. **C**, Four-chamber view MCE of a patient with severe, single vessel, proximal LAD artery stenosis. HFR image demonstrates more extensive and intense perfusion defect within mid to apical infero-septal wall compared with conventional MCE. L indicates left; and R, right.

In this first study in humans with OCAD, HFR MCE, despite using a research platform without optimization and without using a detailed data processing algorithm unlike conventional MCE, is as accurate for the detection of OCAD. However, HFR MCE detected larger and more severe ischemic burden which has diagnostic and prognostic implications even in the absence of OCAD as it may detect perfusion defects due to microvascular dysfunction, although it may compromise specificity for the detection of OCAD. If established through larger trials, HFR MCE compared with contemporary techniques, by virtue of its improved accuracy, safety, and relatively low cost, may well become the initial functional test for the assessment of OCAD.

## ARTICLE INFORMATION

### Sources of Funding

None.

### Disclosures

Dr Senior has received speaker fees from Bracco, Milan, Italy, Lantheus Medical Imaging, Boston, Massachusetts, USA, GE healthcare, St Giles, Amersham, United Kingdom, Philips healthcare, Eindhoven, Holland. The other authors report no conflicts.
